# Effects of Apelin on Cardiovascular Aging

**DOI:** 10.3389/fphys.2017.01035

**Published:** 2017-12-12

**Authors:** Ying Zhou, Yong Wang, Shubin Qiao, Liang Yin

**Affiliations:** ^1^Department of Cardiology, China-Japan Friendship Hospital, Beijing, China; ^2^Department of Cardiology, Cardiovascular Institute of Fuwai Hospital, Chinese Academy of Medical Sciences, Peking Union Medical College, Beijing, China; ^3^School of Science, Beijing University of Chemical Technology, Beijing, China

**Keywords:** Apelin, cardiovascular diseases, aging, RAAS, Atherosclerosis/CAD

## Abstract

Apelin is the endogenous ligand of APJ, the orphan G protein-coupled receptor. The apelin–APJ signal transduction pathway is widely expressed in the cardiovascular system and is an important factor in cardiovascular homeostasis. This signal transduction pathway has long been related to diseases with high morbidity in the elderly, such as atherosclerosis, coronary atherosclerotic heart disease, hypertension, calcific aortic valve disease, heart failure and atrial fibrillation. In this review, we discuss the apelin–APJ signal transduction pathway related to age-associated cardiovascular diseases.

## Introduction

Apelin was discovered in 1998 as the endogenous ligand of APJ, the orphan G protein-coupled receptor (O'Dowd et al., 1993; Tatemoto et al., 1998). The gene for the APJ receptor has high sequence homology to the angiotensin receptor ATR (O'Dowd et al., 1993; Tatemoto et al., 1998). The preprotein of apelin is a 77-amino acid that is sequentially decomposed by an angiotensin-converting enzyme into four active peptides, i.e., apelin-13, apelin-12, apelin-17, and apelin-36 (Tatemoto et al., 1998; Habata et al., 1999; Hosoya et al., 2000; Lee et al., 2000); among these, the most potent peptide that has the primary active biological function is apelin-13 (Kawamata et al., 2001; Tatemoto et al., 2001).

Aging is one of the primary risk factors in cardiovascular diseases (CVDs) (Dai et al., 2012). Studies have found that the renin-angiotensin system was related to cardiovascular aging. The renin-angiotensin system is one of the major signaling pathways related to the progress of the chronic proinflammatory profile within aged arteries (Wang et al., 2014). Ang II increased markedly in the thickened intima of rats, nonhuman and human primates (Wang et al., 2003, 2005, 2007; Fu et al., 2009). The Ang II receptor, AT1, is upregulated in aged arterial walls (Wang et al., 2005, 2007, 2010).

Ang II was also found to be related to structural, functional, and molecular changes that were found in the hearts of aged animals (Groban et al., 2006; Dai et al., 2009). Ang II levels increased significantly with age in myocardial tissue. Inhibition of Ang II signaling by either angiotensin-converting enzyme inhibitor or angiotensin receptor type II inhibitor was found to slow the progress of age-related cardiovascular changes, providing evidence for the role of Ang II and the effect of RAAS inhibitor in cardiovascular disease in aged people (Basso et al., 2007). Angiotensin-converting enzyme inhibitor and angiotensin receptor type II inhibitor have been shown to inhibit myocardial fibrosis and fibrosis-related arrhythmias in aged mice (Stein et al., 2010).

Because the gene for the APJ receptor has high sequence homology to the angiotensin receptor ATR, many studies concerning apelin–APJ in age-related cardiovascular diseases have been performed. Diseases that are prominent in the elderly, such as atherosclerosis, hypertension, coronary atherosclerotic heart diseases, heart failure, atrial fibrillation and calcific aortic valve disease (CAVD), have been associated with the apelin–APJ signaling system. This review will focus on the apelin–APJ signaling system related to age-associated cardiovascular diseases.

## Apelin/APJ cellular signaling pathways in the cardiovascular system

A number of studies have indicated that the apelin–APJ system is a powerful factor in the cardiovascular system in addition to Angiotension II and ATR. In the cardiovascular system, apelin binds to the APJ receptor on endothelial cells, vascular smooth muscle cells, and cardiac myocytes. As a result, vasodilatation (Reaux et al., 2001) and cardiac inotropic effect are performed (Dai et al., 2006; Yu et al., 2014). Previous studies showed that apelin could inhibit cardiac fibrosis via the prevention of cardiac fibroblast activation and collagen production (Pchejetski et al., 2012).

## Endothelial cells

It was found that apelin could act as a vasodilator in the presence of NO and endothelium (Tatemoto et al., 2001). *In vitro* studies showed that apelin caused NO-dependent vasodilation in human mesenteric arteries (Jia et al., 2007). However, apelin-13 may conduct vasoconstriction and deteriorate hypertension in rats after harming the vascular endothelium (Han et al., 2013).

## Vascular smooth muscle cells

Recent studies showed that apelin-13 could induce vascular smooth muscle cell (VSMC) proliferation by upregulating the expression of Cyclin D1 (Li et al., 2013a). Cui et al. found that apelin prominently reduces apoptosis of human VSMCs; apoptosis was induced by serum deprivation (Cui et al., 2010). Wang et al. determined that apelin promotes VSMC migration through a PI3K/Akt/FoxO3a/MMP-2 pathway (Wang et al., 2015).

## Cardiomyocytes

The cardiac inotropic effect of apelin has been found in recent studies. Apelin showed direct effects on the contractility of cardiomyocytes. Apelin significantly improved sarcomere shortening in normal and failing cardiomyocytes. One of the mechanisms may be an increased myofilament sensitivity to Ca(2+), because apelin enhanced the activity of the Na(+)/H(+) exchanger with consequent intracellular alkalinization (Farkasfalvi et al., 2007). Isolated left ventricular cardiomyocytes lacking either apelin or APJ show less sarcomeric shortening and a decreased velocity of contraction (Charo et al., 2009).

## Apelin and aging-related cardiovascular diseases

### Apelin and atherosclerosis

The most important part in atherosclerotic progress is atherosclerotic plaque formation. Angiotensin had been proved to be an atherosclerosis inducer, so it is hypothesized that apelin is also a critical factor in the progress of atherosclerosis (Li et al., 2010). Pitkin SL et al. found that apelin was upregulated in human atherosclerotic coronary arteries and is also localized to the plaque, co-localizing with markers for macrophages and smooth muscle cells (Pitkin et al., 2010). Chun et al. (2008) found that apelin downregulated AS formation by inhibiting AngII actions in mice. However, Hashimoto et al. (2007) found that apelin can promote AS by mediating oxidative stress-related AS in vascular tissue. Although it is clear that apelin is an important factor for AS, it is still difficult to define whether apelin/APJ has a beneficial or harmful role in atherosclerosis. The contribution of apelin in the development of AS remains to be determined.

### Apelin and cardiac atherosclerotic diseases

Angiogenesis is one of the most important mechanisms of myocardial repair for cardiac atherosclerotic diseases, such as myocardial infarctions (MI) and ischemic heart diseases. The effect of apelin in angiogenesis in animal models of AMI and ischemic heart disease have been demonstrated with positive results (Li et al., 2007; Mao et al., 2011). It was reported that apelin decreased in patients with MI, and a lower apelin level was associated with downregulated myocardial angiogenesis (Li et al., 2010). Injection of apelin into the ischemic myocardium stimulated neovascularization in the peri-infarct area through paracrine activity (Tempel et al., 2012). Li et al. (2008) found that apelin-13 could promote myocardial angiogenesis, inhibit cardiac fibrosis, attenuate cardiac hypertrophy, and improve cardiac function at 14 days after myocardial infarction. Regarding the mechanism for apelin-13 promoting angiogenesis after myocardial infarction, studies explored that apelin could upregulate the expression of SDF-1a/CXCR-4 and the homing of vascular progenitor cells (Wang et al., 2013). To confirm the angiogenesis effect of apelin in the heart, a further study was performed in which murine bone marrow cells were pretreated by apelin and later delivered into myocardium. As a result, myocardial angiogenesis increased and cardiac fibrosis was attenuated (Kidoya et al., 2010).

Because myocardial angiogenesis plays an important role in cardiac function in cardiac atherosclerotic diseases, the positive effect of apelin indicates that it could be used as a myocardial protecting factor after myocardial infarction. Further clinical studies are needed to confirm this effect of apelin.

### Apelin and hypertension

Hypertension is highly related to endothelial dysfunction and arterial stiffness. In healthy individuals, age is an essential factor in arterial structure and function alteration (Azizi et al., 2013). Increases in arterial stiffness are mostly attributed to aging-induced endothelial dysfunction (Arnett et al., 1994; Blacher et al., 1999; Li et al., 2012, 2013b). NO plays an important role in vasodilation (Laurent et al., 2001). Aging is associated with the impairment of arterial eNOS mRNA and protein expression, which contribute to increased arterial stiffness and elevated blood pressure (Csiszar et al., 2002; LeBlanc et al., 2008; Donato et al., 2009; Novella et al., 2013).

Apelin administration caused a powerful antihypertensive effect in normal and hypertensive animal models (Rowe, 1987; Katugampola et al., 2001; Napoli and Ignarro, 2001). Administration of apelin to patients causes NO-mediated arterial vasodilation with no significant effect on peripheral venous tonus (Japp et al., 2008; Quazi et al., 2009). This antihypertensive effect was blocked in the co-presence of NOS inhibitor, indicating that apelin leads to vasodilation through a mechanism associated with NO (Szokodi et al., 2002). The antihypertensive effect of apelin was inhibited, and at the same time, the eNOS phosphorylation in the endothelial cells was downregulated in APJ-deficient mice (Zhang et al., 2006). Therefore, reductions of NO expression may be associated with reduced plasma apelin levels in the elderly and may result in endothelial dysfunction and arterial stiffness. Moreover, the concentration-dependent vasodilatation effect of apelin was normal in endothelium-intact mammary arteries but disappeared after endothelial removal, indicating that the antihypertensive effect of apelin is endothelium-dependent (Charles et al., 2006; Maguire et al., 2009).

Future research about the effect of apelin in patients with hypertension should focus on the mechanism in addition to the NO pathway in order to find hidden side effects of apelin in patients with hypertension in further clinical studies.

### Apelin and heart failure

Because it was demonstrated that apelin had a potent inotropic effect in myocardial cells, further *in vivo* studies were performed to find the effect of apelin in heart failure. Both myocardial and plasma apelin levels of heart failure patients decreased simultaneously, suggesting that the heart is a major source of circulating apelin; it plays an essential role in the maintenance of myocardial systolic function (Dalzell et al., 2015). Several studies focused on aged animals and humans with heart failure. Compared with control aged mice, apelin^−/−^ mice have an increased risk of progressive left ventricular systolic dysfunction with age (Lee et al., 2005). Infusion of apelin-13 in aged apelin^−/−^ mice could improve left ventricular systolic dysfunction (Ishida et al., 2004). In humans, plasma apelin levels decreased in advanced heart failure in most studies, but the studies that focused on the early stages of heart failure demonstrated that apelin levels remained normal or even increased in early stages (Chen et al., 2003; Kuba et al., 2007; Miettinen et al., 2007; Japp et al., 2010). A study from Pitkin SL et al. may explain this phenomenon. They found that apelin receptor APJ's density significantly decreased in the left ventricle of patients with dilated cardiomyopathy or ischemic heart disease compared with that in the left ventricle of control patients, but apelin peptide levels remained unchanged. The decrease in receptor density in heart failure may limit the positive inotropic actions of apelin, resulting in an initial compensatory mechanism by increasing apelin to improve myocardial contractility (Pitkin et al., 2010). Serum apelin levels were upregulated after cardiac resynchronization therapy together with an improvement in myocardial systolic function (Földes et al., 2003). The administration of apelin in patients with heart failure led to the improvement of cardiac output and vasodilatation (Chong et al., 2006).

Thus, apelin could be used as a factor that has both a cardiotonic and afterload lowering effect in heart failure patients. It seems that apelin has a similar effect to that of BNP in heart failure, so future clinical studies could be designed to compare these two factors, because Nesiritide's effect has been confirmed.

### Apelin and atrial fibrillation (AF)

The expression of apelin in normal atrial myocardium of humans is extremely high (Miettinen et al., 2007). Compared with control subjects with sinus rhythm, patients with atrial fibrillation had significantly lower plasma apelin levels (Francia et al., 2007). Another study showed that if patients could remain in sinus rhythm, the circulating apelin level would rise subsequently as a result (Ellinor et al., 2006). Atrial fibrillation will lead to the loss of atrial systolic function and atrial tissue remodeling. It may be deduced that downregulation of atrial apelin synthesis is a result of increased atrial diastolic filling pressures in patients with atrial fibrillation. Moreover, it has been shown that apelin significantly changes atrial electrophysiology with a shortening of action potential duration that may be caused by its effects on multiple ionic currents (Cheng et al., 2013).

The morbidity of atrial fibrillation increases with age in humans. Based on the existing studies concerning apelin and atrial fibrillation, the level of apelin in patients with AF may reflect the systolic function of the atrium. Further studies could focus on the predictive effect of apelin in the morbidity of atrial fibrillation and the possibility of maintaining a sinus rhythm.

### Apelin and calcific aortic valve disease

Aortic stenosis and calcific aortic valve disease (CAVD) are leading valvular heart diseases in the elderly (Kallergis et al., 2010). The prevalence of aortic stenosis is only approximately 0.2% in adults over 50 years of age but increases to 9.8% for adults over 80 years of age (Otto and Prendergast, 2014). In tissues of stenotic aortic valves, the expression levels of both mRNA and protein of apelin increased (Nishimura et al., 2014). The levels of apelin and its receptor APJ are upregulated in patients with calcified aortic valve stenosis (Peltonen et al., 2009). Apelin may be upregulated compensatorily in the development of aortic valve stenosis. APJ receptor antagonists might be beneficial in the treatment of aortic valve stenosis by suppressing angiogenesis, osteoblast activity and collagen synthesis (Peltonen et al., 2009).

## Conclusion

CVDs are the most common causes of death in most countries of the world, and old age is a risk factor for CVDs. Studies have found that RAAS plays an important role in cardiac aging. As the newest member in the RAAS system, it has been shown that apelin can increase cardiac contractility, lower blood pressure, increase atherosclerotic plaque stability and ameliorate the harmful effects of AT1 receptor activation in the progression of aortic valve stenosis. Further clinical trials are necessary to study the application of apelin in the treatment of cardiac aging, hypertensive cardiomyopathy, and heart failure.

## Author contributions

YZ is the main author of the article. YW, SQ, and LY revised the article.

### Conflict of interest statement

The authors declare that the research was conducted in the absence of any commercial or financial relationships that could be construed as a potential conflict of interest.
